# Laser treatments as an adjunct to non-surgical periodontal therapy in subjects with periodontitis and type 2 diabetes mellitus: a systematic review and meta-analysis

**DOI:** 10.1007/s00784-023-04873-y

**Published:** 2023-02-28

**Authors:** Stefano Corbella, Elena Calciolari, Nikolaos Donos, Alice Alberti, Pinar Ercal, Luca Francetti

**Affiliations:** 1 Department of Biomedical, Surgical and Dental Sciences, Università degli Studi di Milano, Milan, Italy; 2 IRCCS Istituto Ortopedico Galeazzi, Milan, Italy; 3 Centre for Oral Clinical Research Institute of Dentistry, Barts and The London, School of Medicine and Dentistry, Queen Mary University of London, London, UK; 4 Department of Medicine and Surgery, Centre of Dentistry, University of Parma, Parma, Italy

**Keywords:** Antimicrobial photodynamic therapy, Diabetes mellitus, Laser, Non-surgical periodontal treatment, Periodontitis, Systematic review

## Abstract

**Objectives:**

Periodontal disease and diabetes have an extensively investigated bidirectional correlation. Non-surgical periodontal treatment (NSPT) was proven to contribute to glycemic control. Moreover, it may benefit from the association of adjunctive therapies. The aim of the present systematic review is to assess the clinical efficacy of NSPT in association with laser (LT) or photodynamic therapy (PDT) in controlled or uncontrolled diabetic patients, and to grade the level of evidence.

**Materials and methods:**

Randomized controlled clinical trials with at least 3-month follow-up were searched in MEDLINE via OVID, EMBASE, and Cochrane Central, screened for inclusion, and grouped based on the performed treatments, follow-up time, type of diabetes, and level of glycemic control.

**Results:**

Eleven RCTs with 504 total subjects were included. The adjunct of PDT showed a statistically significant 6-month difference in PD changes (with low certainty of evidence), but not in CAL changes, while a significant difference in 3-month PD and CAL changes was found with the adjunct of LT (low certainty of evidence). Patients treated with PDT registered a higher decrease in HbA1c levels at 3 months, but no significant difference was noted at 6 months; LT also led to better HbA1c changes at 3 months with a moderate certainty of evidence.

**Conclusion:**

Despite the promising short-term HbA1c decrease, the results should be interpreted with caution due to the small effect sizes and the statistical heterogeneity, and further evidence from well-designed RCTs is needed to support the routine use of PDT or LT in adjunct to NSPT.

## Introduction

The bidirectional relationship between hyperglycemia (all types of diabetes) and periodontitis is well-known and widely documented in the scientific literature [1]. Several recent studies confirmed that diabetes represents a significant independent risk factor, it influences oral health in general, and it is a known cause of increased tooth loss rate [2–4]. Indeed, diabetes is considered one of the major risk factors for periodontal diseases, being the risk of having periodontitis in subjects with diabetes approximately three-fold higher than in healthy subjects [5].

Several mechanisms were pointed out to explain the linkage between diabetes mellitus and periodontitis. In general, diabetes can trigger an increase of the inflammatory response towards the oral microbiota (e.g., augmenting IL-1, IL-6, TNF-α) and can impair the immune host response, thus creating favorable conditions for the development and worsening of periodontal diseases in predisposed subjects [6, 7].

At the same time, periodontitis is responsible of increasing insulin resistance and may enhance the risk for diabetes or promote an impairment of glucose tolerance mechanisms. Based on the existing literature, there is evidence that periodontitis could be associated with an increased incidence of diabetes in specific cohorts of systemically compromised patients [8], as well as in the general population, since people with normal glycemic control and periodontitis are more prone to develop diabetes than periodontally healthy subjects [9]. Moreover, periodontitis represents an independent risk factor for microvascular complications in diabetic subjects, such as nephropathy, neuropathy, and retinopathy [10]. The biological plausibility of a correlation between periodontitis and diabetes finds a substantial support considering the low-grade inflammatory systemic status that is induced by periodontitis itself, which could be the basis of an increased susceptibility to diabetes in particularly predisposed subjects [11, 12]. Furthermore, periodontitis-induced systemic inflammation could also contribute to hematopoiesis by increasing the production of myeloid cells that are more responsive to inflammation, and this process might potentially be at the basis of different comorbidities [13].

Given the bidirectional correlation between diabetes and periodontitis, it was demonstrated that non-surgical periodontal treatment (NSPT) in subjects with periodontitis and diabetes could influence glycemic control [14–16]. A recent Cochrane systematic review, including 35 studies and accounting for a total of 3249 participants, found a reduction of HbA1c of 0.43% at 3–4 months after non-surgical treatment (any type of subgingival instrumentation), thus suggesting that periodontal therapy contributes to glycemic control [15].

Despite NSPT is considered to be generally effective in the treatment of periodontitis, we expect that a certain number of pockets (about 26% at 6/8 months) will not close because of local factors (e.g., depth of initial pocket, anatomy of the tooth and of the defect) and factors related to the patient (e.g., smoking, systemic diseases, compliance with oral hygiene) or operator (ability to successfully remove the deposits and to motivate the patient) [17]. Therefore, adjunctive measures that could enhance the outcomes of NSPT have been proposed [18–22]. Among these adjunctive therapies, the systematic review published by Salvi and coworkers, considered in the recently published S3-level treatment guideline of the European Federation of Periodontology, examined the efficacy of laser (LT) and photodynamic therapy (PDT) [20]. While the authors did not find differences when focusing on systemically healthy periodontitis patients, a specific analysis of the effects of laser or PDT in a particular susceptible group of subjects, such as diabetic patients, considering both periodontal and glycemic outcomes, is still missing. It might be hypothesized that LT and PDT, due to their anti-inflammatory effect and the ability of modulating the inflammatory response in other systemic clinical conditions [23], can be a valuable adjunctive therapy for the treatment of diabetic periodontitis patients. Moreover, the differences in the subgingival population that exist between diabetic and non-diabetic periodontal patients could be a further reason for the need of different/additional approaches for treating the periodontal disease in diabetic patients [24]. Despite some systematic reviews with heterogeneous methodology are available in this field [25, 26], no meta-analysis and critical appraisal of certainty of evidence have been published comparing PDT/LT as an adjunct to NSPT to NSPT alone. Moreover, the previously published studies reported inconclusive results.

There is therefore the need of systemically addressing the evidence about adjunctive periodontal treatments such as PDT and LT in subjects with diabetes, mainly because of the high prevalence of the disease and the need of considering the effect of this systemic disease on treatment outcome in studies designed for this specific purpose.

The present systematic review of the literature aimed to fill this knowledge gap and to assess the efficacy of NSPT performed with the adjunct of LT or PDT in patients with type II diabetes mellitus and to grade the level of available evidence.

## Materials and methods

The protocol of the study was registered in PROSPERO database (number CRD42021237742) before study initiation. The protocol followed the instructions provided by the Cochrane Handbook for Systematic Review of Interventions – Second Edition [27].

The aim of this review was to answer the following focused question: in periodontitis patients affected by type II diabetes mellitus, what is the efficacy of PDT and LT as an adjunct to non-surgical periodontal therapy in terms of pocket closure, probing pocket depth (PPD) reduction, and clinical attachment level (CAL) gain?

### Eligibility criteria

The criteria for considering studies for this review based on the PICOS are:

-  Population (P): ≥ 18 years old, previously untreated periodontitis patients (defined following the current and past classifications [28, 29] as stage II, stage III, or stage IV periodontitis (any grade) or moderate to severe periodontitis) affected by controlled or uncontrolled type II diabetes (T2DM) (code 5A11 following the International Classification of Diseases of the World Health Organization [30]), defined as presence of insulin resistance [31].
- Intervention (I): (a) Physical treatment (e.g., LT, PDT) as an adjunct to non-surgical treatment (sub-gingival instrumentation) of periodontitis.
-  Control (C): The same non-surgical treatment of periodontitis associated with placebo or without adjunctive therapy, or performed according to a different protocol.
-  Outcomes (O):
  Primary outcomes:
  - Proportion or number of pockets closed (defined as PPD < 5 mm and no bleeding on probing (BOP)); reduction in PPD, which is defined as the distance from the gingival margin to the base of the pocket as assessed with a standardized (UNC-15) periodontal probe with a force of 0.2/0.25N; changes in CAL, which is the measurement of the position of the soft tissue in relation to cemento-enamel junction (CEJ).
  Secondary outcomes:
  - Site-specific response to subgingival instrumentation (in horizontal defects, intrabony defects and furcations)
  - Changes in HbA1c levels
  - Changes in BOP or gingival inflammation and in plaque levels
  - Number of teeth lost or extracted during the examination period
  - Patient-reported outcome measures (PROMs), including adverse events
- Studies (S): Randomized controlled clinical trials with at least 3-month follow-up. Split-mouth studies were excluded due to the risk of carry-over effects

### Search and study selection

The electronic search for pertinent articles was performed searching the following databases: MEDLINE via OVID, EMBASE, and Cochrane Central and by using the search strategy presented in Appendix 1. Grey literature was searched for pertinent articles interrogating Greylit and OpenGrey. Trials registers (ClinicalTrials.gov and EU Clinical Trials Register) were also searched through keywords. A manual search was performed for all the issues published since 1990 of the following journals: *Journal of Clinical Periodontology*, *Journal of Periodontology*, *Journal of Periodontal Research*, *Journal of Dentistry*, and *Journal of Dental Research*. Besides checking the reference list of all included papers, Scopus was consulted to check the articles citing the papers included. No language limitations were posed. Conference papers and abstracts were excluded.

The last electronic search was performed in all databases on 10 February 2022.

Two reviewers (SC, EC) independently screened titles and abstract for preliminary check of inclusion criteria (1st stage). The second stage of articles selection was performed by the same reviewers, by carefully screening the full texts of the papers retrieved after preliminary check. In case of disagreement, a third reviewer (ND) was interrogated to solve the dispute. Reasons for exclusion in the second step were recorded, and the level of concordance in each step of the selection process was assessed through Cohen’s kappa.

### Data extraction

The process of data extraction was performed independently by two authors (AA, PE) who retrieved the following information from the included studies: authors’ names, year of publication, country, characteristics of the sample (age distribution, sex distribution, ethnicity, educational status, smoking status), characteristics of diabetes (definition and type, level of control of the disease, HbA1c levels, drugs), definition/assessment of periodontitis, characteristics of the periodontal treatment and of the adjunctive physical therapy, clinical data before and after the treatment (number of teeth lost, proportion of closed periodontal pockets, mean periodontal probing depth (PD), mean CAL, gingival bleeding indexes (gingival bleeding index, gingival index (GI), percentage of bleeding sites (BOP), plaque indexes (plaque index (PI), Turesky-modified plaque index, proportion of sites with visible plaque) or difference between baseline and follow-up values, occurrence of adverse events or complications, and patients’ reported outcomes (PROMs).

In case of missing/unclear information, an attempt was made to contact the authors by email.

### Risk of bias evaluation and quality of evidence assessment

The risk of bias evaluation and the quality of evidence assessment were performed independently by two reviewers (SC, LF) and any disagreement resolved by discussion.

The criteria for evaluating the risk of bias in the included studies were the ones of the Cochrane risk-of-bias tool for randomized trials 2.0 [27]:

- Bias arising from the randomization process
- Bias due to deviations from intended interventions
- Bias due to missing outcome data
- Bias in measurement of the outcome
- Bias in selection of the reported result

The overall risk-of-bias judgment was considered as *high risk* if the level of risk of bias was high for at least one domain or if the trial was judged to have some concerns for multiple domains (three). If the trial was judged to have some concerns for less than three domains, the overall risk of bias was “some concerns,” while the study had *low risk* of bias if all domains were judged to have low risk.

The funding bias was estimated by evaluating if authors disclosed their potential sources of competing conflict of interest and the source of funding for the studies they carried on (if any).

The quality of the available evidence was assessed for each comparison and for each outcome in the meta-analysis by using the Grading of Recommendations, Assessment, Development and Evaluations (GRADE) approach [32]. GRADE provides a system for rating quality of evidence and strength of recommendations that is explicit, comprehensive, transparent, and pragmatic.

### Summary measures and synthesis of the results

In order to perform the meta-analysis, studies were grouped based on the treatments that were carried out, follow-up time, and, whenever possible, based on the type of diabetes and on level of control. In particular, we distinguished between photodynamic therapy (PDT) and direct laser application (LT). Meta-analysis was performed by using the software RevMan (Review Manager Version 5.3, 2014; The Nordic Cochrane Center, The Cochrane Collaboration, Copenhagen, Denmark) if at least three papers were available for each comparison.

For each continuous outcome, the difference between baseline and follow-up values was extracted with its specific error measure (standard deviation, standard error, or variance). When difference values were not reported, they were calculated as the difference between baseline and follow-up values and error (namely, standard deviation) was computed following the procedure described in Appendix 2. In the meta-analysis, the effect size was computed through the weighted mean method, and results were combined using the DerSimonian and Laird’s random-effect model [33], assuming heterogeneity among studies. Cochran’s test served to measure the consistency of the results, considering it significant if *P* < 0.1. *I*^2^ statistics was applied to measure heterogeneity (total variation across studies that was due to heterogeneity rather than to chance) [27].

Regression meta-analysis was performed to evaluate the effect of baseline HbA1c% on the primary outcome measures.

Small study effects, as proxy for publication bias, were assessed by testing for funnel plot asymmetry and by calculating Egger’s bias, as described in the Cochrane Handbook [27].

## Results

The results of this systematic review are herein presented following the Preferred Reporting Items for Systematic Reviews and Meta-Analysis (PRISMA) guidelines [34].

**Fig. 1:**
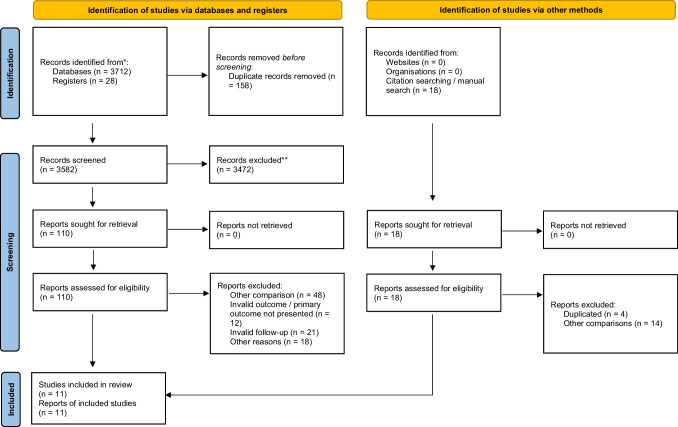
PRISMA diagram of article selection process

The summary of the article selection process is summarized in Fig. 1. Eleven RCTs were included in the analysis [35–45], which accounted for a total of 504 subjects, examined with a follow-up ranging from 1 to 6 months.

In particular, seven papers compared NSPT to NSPT and adjunctive PDT in subjects with diabetes [35–37, 40, 42, 43, 45]. In all the studies, in the test groups, non-thermal diode laser was used to irradiate a photosensitizer agent. In one study, NSPT was performed following a “Full-mouth disinfection” protocol in both groups [43].

Four studies compared NSPT to NSPT and adjunctive DL use (with settings varying between 0.8 and 1.8 W) in subjects with diabetes [38, 39, 41, 44]. In all studies, the control groups were treated according to a quadrant-based NSPT protocol. In four studies, the periodontal disease was classified following the 2017 classification [28], including stage II, stage III, and stage IV periodontitis and grade B or C [36, 40, 44, 45]. The other included studies used older classifications and diagnostic parameters [46].

Considering the characteristics of the population, three studies were performed in Saudi Arabia [35, 36, 40], three in Brazil [37, 42, 45], two in India [38, 44], two in Turkey [39, 41], and one in Pakistan [43]. In all studies, only T2DM was considered, with different level of controls defined on the basis of HbA1c: three studies included patients with HbA1c > 7% [39, 42, 45]; one included subjects with HbA1c > 6% [44]; one considered HbA1c ≥ 6.5% [43]; one < 7% [37]; and in one study, subjects with HbA1c between 5.7 and 8.5% were included [41], while other studies adopted different definitions [35, 36, 38, 40]. One study clearly stated that only subjects with decompensated T2DM were included [45], while in four studies, patients with major diabetic complications were excluded [35, 39, 42, 43]. Smokers were excluded in all studies.

Additional details about the characteristics of the studies are shown in Table 1.

**Table 1:** Main characteristics of the included studies

Authors	Study type	Number of subjects; sex (m/f)	Age (mean ± SD (range))	Systemic conditions/health status	Ethnicity	Periodontal disease	Diabetes	Outcomes	Follow up	Type of probe and n sites/tooth evaluated	Group 1	Group 2	Group 3	Group 4
Al-Zahrani et al. 2009 Saudi Arabia	RCT, single-blind	43; 17/26 G1: 7/8 G2: 4/10 G3: 6/8	52.21 ± 8.35 G1: 53.14 ± 10.91 G2: 51.42 ± 6.24 G3: 51.92 ± 7.28	Excluded: atb in the previous 6 mo, pregnancy	NS	CAL loss ≥ 3 mm at ≥ 30% of sites	T2DM, no major diabetic complications	PD, CAL, REC, plaque and bleeding scores, HbA1c	12 wks	6 sites per tooth	SRP	SRP + doxycycline 2× 100 mg for day 1 and then 100 mg once a day for 13 days *not considered in the present review	SRP + PDT (670-nm non-thermal diode laser)	-
Koçak et al. 2016 Turkey	Parallel RCT, single-blind	60; 30/30 G1 15/15 G2 15/15	35–60 G1 53.1 ± 5.1 G2 51.7 ± 5.2	Excluded: other systemic diseases, smoking, alcoholism, atb in the previous 6 mo, immunosuppressive medications, pregnancy/lactation	NS	CP, 8 ≤ sites with PD ≥ 5 mm, ≥ 17 remaining teeth	T2DM, no changes in diabetes therapy in the previous 12 mo HbA1c 5.7–8.5%	PD, CAL, GI, PI, HbA1c, GCF levels of IL-1β, IL-6, IL-8, ICAM, VCAM	1 and 3 mo	PCP-UNC 15 PD/CAL: 6 sites per tooth for, GI/PI: 4 sites per tooth	SRP	SRP + diode laser (940-nm, indium–gallium–aluminum–phosphate diode laser)	-	-
Barbosa et al. 2018 Brazil	RCT, Pilot study	12; 4/8	52.2	Excluded: other systemic disease influencing periodontal status, smoking, atb in the previous 3 mo, pregnancy	NS	Moderate to severe periodontitis	T2DM using oral hypoglycemic agents and/or insulin and who had glycated hemoglobin (HbA1c) values below 7% measured no more than 90 days prior to selection were included in the study	PD, CAL, PI, GBP, GSP, HbA1c	30, 90, 180 d	Williams color-coded probe	SRP+ aPDT (660-nm diode laser)	SRP	-	-
Chandra et al. 2019 India	RCT, single-blind	36; 18/18 9/9; 9/9	48/50.6	Excluded: smoking, alcoholism, pregnancy/lactation	NS	Generalized CP, PPD 4–7 mm with CAL ≥ 2 mm, or greater and each quadrant having at least 3 teeth (≥ 3 in each quadrant)	T2DM, non insulin dependent	PD, CAL, PI, GI, microbiological Aa, Pg, HbA1c	3 mo	UNC-15	SRP + diode laser (808 nm and a power setting of 1.5–1.8W were used in continuous, contact mode with a thin flexible fiber optic cable (320 nm)) + irrigation with saline	SRP + irrigation with saline	-	-
Dengizek Eltas et al. 2019 Turkey	RCT, single-blind	37; 17/20	49.7/51.85	Excluded: other systemic diseases affecting periodontal status, smoking, atb or anti-inflammatory drugs in the previous 6 mo, pregnancy/lactation	NS	Generalized CP, PD 4-7 mm in ≥4 teeth in the upper jaw, ≥ 20 remaining teeth	T2DM for ≥ 2 yrs, no major diabetes complications HbA1c ≥ 7%	PD, CAL, PI, GI, HbA1c, CRP	3 and 6 mo	PCP-12	SRP + diode laser (810 nm wavelength, 1 W power, contact mode using a 400-μm fiber optic tip)	SRP	-	-
Mirza et al. 2019 Pakistan	RCT	30; 20/10 11/4; 9/6	51.45/52.93	Excluded: current/former smokers, atb in the previous 3 mo, pregnancy/lactation	NS	Mild to moderate periodontits, no periodontal treatment in previous 6 mo	T2DM for ≥ 2 yrs, no major diabetic complications HbA1c ≥ 6.5%	PD, BOP, PI, AL, HbA1c, Advanced glycation end-products in GCF	3 and 6 mo	UNC-15 6 sites per tooth	FMD+ PDT (670 nm 150 nW fluency of 22 J/cm^2^ and density of 1.1 W/cm^2^)	FMD	-	-
Macedo et al. 2013 Brazil	RCT	30; 16/29 G2 6/9 G1 5/10	48.73 ± 7.11 G2 49.4 ± 6.8 G1 48.1 ± 9	excluded: smoking in the previous 5 years, atb in the previous 6 months, pregnancy/lactation	NS	≥ 1 site with PPD ≥ 5 mm on each quadrant, and ≥ 2 teeth with CAL loss ≥ 6 mm	T2DM > 5 yrs, no major diabetic complications HbA1c > 7%	PPD, CAL, PI, BOP, HbA1c, suppuration	3 mo	Computerized periodontal probe 6 sites per tooth	SRP	SRP + aPDT (660-nm diode laser, phenothiazine chloride photosensitizer-induced aPDT)	-	-
Al-Zawawi et al. 2020 Saudi Arabia	RCT	128; diabetic subjects 27/6	diabetic subjects 55.5	Excluded: other systemic diseases, smoking, tobacco chewing, alcoholism, pregnancy/lactation	NS	Stage II grade C periodontitis according to Consensus report 2017 World Workshop	T2DM	PD, CAL, GI, PI, MBL, cortisol in GCF, HbA1c	3 and 6 mo	Click-probe 6 sites per tooth	(Diabetic patients) SRP + aPDT (diode laser at 660 nm and 150 mW, irradiation was performed for 60 s with a fiber optic tip of 300 μm diameter)	(Diabetic patients) SRP	(Non-diabetic patients) SRP + aPDT (diode laser at 660 nm and 150 mW, irradiation was performed for 60 s with a fiber-optic tip of 300 μm diameter) [not included]	(Non-diabetic patients) SRP [not included]
Elsadek et al. 2020 Saudi Arabia	RCT	60; 34/26 11/9; 10/10; 13/7	52.16/51.87/52.88	Excluded: other systemic disease influencing periodontal disease course, current/former smokers, patients on anti-inflammatory/antimicrobials/statin therapy, pregnancy/lactation	NS	Stage III and grade C generalized periodontitis, CAL ≥ 5 mm and radiographic bone loss extending to middle or apical third of root, no previous periodontal therapy	T2DM (ADA 2018)	PD, CAL, REC, BOP, PS, HbA1c	3 mo	UNC probe 6 sites per tooth	SRP + PDT (diode laser670 nm wavelength, 150 mW maximum power, 60 s per site, 20 J/cm^2^ per site)	SRP + probiotic *L. reuteri* (2 × 108 CFU/tablet, 2 lozenges/day for 3 wks)	Debridement	-
Soi et al. 2021 India	RCT	37; 21/16	51.58/51.67	Excluded: other systemic diseases, smoking, alcoholism, medication other than hypoglycemics, pregnancy/lactation	NS	Stage II or III/grade B or C periodontitis, ≥ 8 sites with CAL loss ≥ 3 mm and PPD ≥ 3 mm, ≥ 20 teeth	T2DM (FPG ≥ 126 mg/dl, RBS ≥ 200 mg/dl, PP ≥ 200 mg/dl) HbA1c > 6%	PD, CAL, PI, GI, RBS, FBS, HbA1c	1, 3, 6 mo	UNC-15 CAL, PD: 6 sites per tooth, PI, GI: 4 sites per tooth	SRP + diode laser (0.8 W, pulse interval 1.0 ms, pulse length 1.0 ms, 24 J)	SRP	-	-
Claudio et al. 2021	RCT, double-blind (surgeon and examiner)	34, 31 examined (22/9)	G1: 53.13 ± 7.58 G2: 54 ± 8.56	Age 30–70; excluded: medical disorders that required antibiotic prophylaxis, antibiotics, anti-inflammatories, anticonvulsants, immunosuppressants or calcium channel blockers in the last 6 mo, smokers in the last 12 mo, pregnancy	NS	Periodontitis stages III and IV, grade C with at least 6 sites with PD and CAL ≥ 5 mm and BOP in at least 15 teeth, excluding third molars; no SRP in the last 6 mo	decompensated DM2: HbA1c ≥ 7.0%	PD, CAL, REC, PI, BOP, number of PD ≥ 5 mm, *P. gingivalis* and *P. intermedia* quantification	3 mo, 6 mo	PCPUNC-15, Hu-Friedy, six sites of each tooth	SRP	SRP + aPDT (immediately after SRP, 48 and 96 h after in pockets with PD ≥ 5 mm)	-	-

*Abbreviations:ADA* American Diabetes Association, *atb* antibiotics, *BOP* bleeding on probing, *CAL* clinical attachment level, *CFU* colony-forming units, *CP* chronic periodontitis, *CRP* C-reactive protein, *d* days, *FPG* fasting plasma glucose, *FBS* fasting blood sugar, *GCF* gingival crevicular fluid, *GSP* glycated serum proteins, *GBP* glycated blood proteins, *Hb1Ac* glycated hemoglobin 1Ac, *mo* months, *NS* not specified, *PP* 2-h post-prandial glucose, *PPD* probing pocket depth, *RBS* random blood sugar, *REC* recession, *wks* weeks, *yrs* years, *SRP* scaling and root 22planing, *OHI* oral hygiene instructions, *PDT* p22hotodynamic therapy, *FMD* full-mouth disinfection, *UNC* University of North Carolina, *T2DM* type 2 diabetes mellitus, *Aa Aggregatibacter actynomycetemcomitans, Pg Porphyromonas gingivalis*

### Risk of bias evaluation

**Fig. 2:**
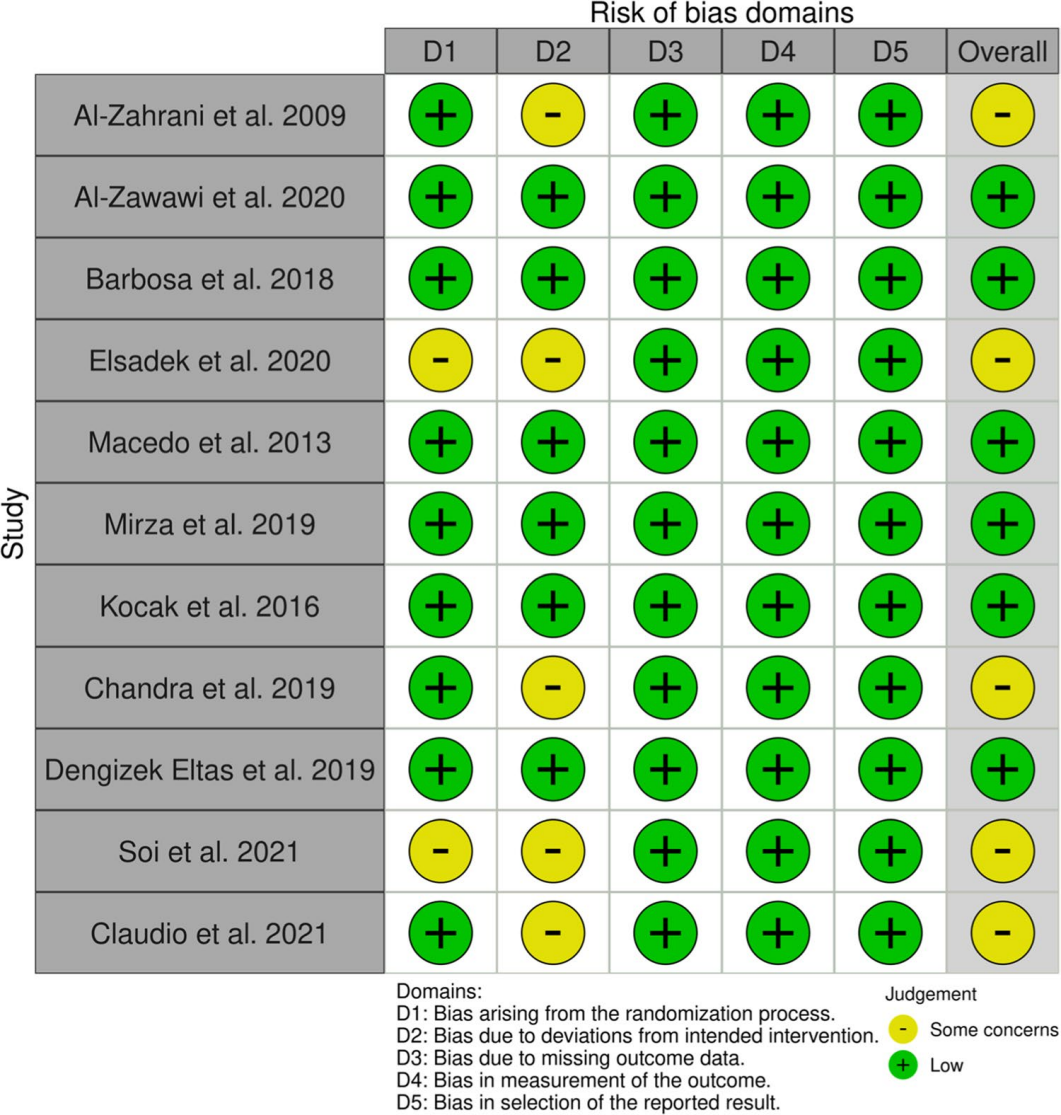
Diagram showing the results of risk of bias evaluation

The results of risk of bias evaluation are reported in Table 2. Five studies raised some concerns about the risk of bias due to the methods of randomization and to the blinding of subjects [35, 38, 40, 44, 45], while six studies were at low risk [36, 37, 39, 41–43] (Fig. 2).

**Table 2:** Results of risk of bias evaluation

Study	Randomization process	Deviations from intended interventions	Missing outcome data	Measurement of the outcome	Selection of the reported result	Overall bias
Al-Zahrani et al. 2009	Low	Some concerns	Low	Low	Low	***Some concerns***
Al-Zawawi et al.	Low	Low	Low	Low	Low	***Low***
Barbosa et al. 2018	Low	Low	Low	Low	Low	***Low***
Elsadek et al. 2020	Some concerns	Some concerns	Low	Low	Low	***Some concerns***
Macedo et al. 2013	Low	Low	Low	Low	Low	***Low***
Mirza et al. 2019	Low	Low	Low	Low	Low	***Low***
Kocak et al. 2016	Low	Low	Low	Low	Low	***Low***
Chandra et al. 2019	Low	Some concerns	Low	Low	Low	***Some concerns***
Dengizek Eltas et al. 2019	Low	Low	Low	Low	Low	***Low***
Soi et al. 2021	Some concerns	Some concerns	Low	Low	Low	***Some concerns***
Claudio et al. 2021	Low	Some concerns	Low	Low	Low	***Some concerns***

#### Synthesis of the results

##### Pocket closure, PD changes, CAL changes

- *NSPT versus NSPT and photodynamic therapy (PDT)*

Meta-analysis based on 4 studies indicated a statistically significant difference in PD changes (favoring the test group) and CAL changes favoring control group 6 months after treatment with a low effect size (PD change: 0.26 mm, CI95%: 0.01, 0.50, *I*^2^: 57%, 137 subjects; CAL change: − 0.2 mm, CI95%: − 0.23, − 0.17, *I*^2^: 0%, 137 subjects) (Table 3).

**Table 3:** Results of the meta-analysis (a positive effect size value means an advantage in test group)

		3 months				6 months			
		*Mean [95% CI] (n° of studies)*	*P*	*I*^2^	*Certainty of evidence (GRADE)*	*Mean [95% CI] (n° of studies)*	*P*	*I*^2^	*Certainty of evidence (GRADE)*
NSPT (with or without placebo) vs NSPT + Photodynamic therapy	*PD red*	0.04 [− 0.13, 0.22] (6)	0.63	93%	Very low	**0.26 [0.01, 0.50] (4)**	**0.04**	**57%**	Low
	*CAL gain*	0.03 [0.00, 0.06] (7)	0.08	0%	Low	− **0.20 [**− **0.23,** − **0.17] (4)**	**< 0.001**	**0%**	Moderate
	*BOP% red*	− **5.95 [**− **9.92,** − **1.98] (5)**	**0.003**	**0%**	Moderate	0.36 [− 9.53, 10.25] (3)	0.94	0%	Low
	*PI red*	0.09 [− 0.08, 0.26] (3)	0.32	79%	Very low				
	*PI% red*	0.23 [− 5.48, 5.95] (5)	0.94	63%	Very low	1.64 [− 3.78, 7.06] (3)	0.55	0%	Very low
	*HbA1c red*	**0.24 [0.17, 0.32] (6)**	**< 0.001**	**14%**	Moderate	− 0.04 [− 0.17, 0.10] (4)	0.62	8%	Low
NSPT (with or without placebo) vs NSPT + diode laser	*PD red*	**0.59 [0.41, 0.76] (4)**	**< 0.001**	**80%**	Low				
	*CAL gain*	**0.84 [0.09, 1.59] (3)**	**0.03**	**86%**	Low				
	*GI red*	**0.34 [0.21, 0.47] (3)**	**< 0.001**	**0%**	Moderate				
	*PI red*	0.21 [− 0.01, 0.43] (3)	0.06	40%	Very low				
	*HbA1c red*	**0.18 [0.07, 0.28] (4)**	**< 0.001**	**0%**	Moderate				

In bold the effect sizes that resulted statistically significant

Three studies reported data about the changes in pockets ≥ 5mm, but they could not be pooled in a meta-analysis because one study reported the mean number of pockets per patient [45] and the others presented the proportions [35, 40]. More specifically, at 3 months, Al-Zahrani and colleagues found a non-significant decrease in the proportion of sites with PD ≥ 5 mm from 11% ± 8% to 6% ± 7% in the test group and from 14% ± 14% to 8% ± 13% in the control group [35]. Likewise, Elsadek et al. [40] indicated a non-significant significant decrease after 3 months in the proportion of sites with PD ≥ 5 mm in both groups (from 12% ± 7% to 4% ± 6% in the test group and from 15% ± 15% to 9% ± 12% in control group). A more recent study reported a decrease in the number of pockets that was significant in both groups after 3 and 6 months from the treatment without a significant intergroup difference [45].

- *NSPT versus NSPT + diode laser (DL)*

Meta-analysis was performed for PD and CAL change at 3 months post-treatment, and it involved 4 and 3 studies, respectively. As reported in Table 3, a significant difference in 3-month PD and CAL change is found when DL was applied as an adjunctive therapy (PD change: 0.59 mm, CI95%: 0.41 mm, 0.76 mm, *I*^2^: 80%, 170 subjects; CAL change: 0.84 mm, CI95%: 0.09 mm; 1.59 mm, *I*^2^: 86%, 112 subjects).

None of the studies reported data on pocket closure. Regression meta-analysis did not reveal any significant effect of baseline HbA1c% on the examined outcomes.

##### Secondary outcomes

- *NSPT versus NSPT and photodynamic therapy (PDT)*

Based on 5 studies, meta-analysis indicated a statistically significant difference in 3-month BoP change between test and control groups (− 5.95% [− 9.92%, − 1.98%]), favoring the latter. However, at 6 months, this difference was no longer significant.

Remarkably, HbA1c decreased significantly more in the test groups than in control groups 3 months after the treatment (0.24, CI95%: 0.17, 0.32), but this outcome was not confirmed at 6 months.

No significant differences were suggested for PI changes (Table 3).

No patient-reported outcomes were reported.

- *NSPT versus NSPT + diode laser (DL)*

The quantitative synthesis based on the data from 3 studies indicated a significant difference, in 3-month GI changes (0.34, CI95%: 0.21, 0.47) favoring the test groups (Table 3). Likewise, the adjunctive use of DL led to better HbA1c changes (0.18, CI95%: 0.07, 0.28) at 3 months (Table 3).

No patient-reported outcomes were reported.

##### Certainty of evidence

The results of the evaluation of the certainty of evidence are summarized in Table 3. Regarding the primary outcomes, in the comparison between NSPT and NSPT + PDT, the certainty was moderate for CAL reduction (6 months) and low for PD changes, while for the comparison between NSPT and NSPT + DL, the certainty of evidence was low. Regarding the secondary outcomes, the adjunctive use of PDT was associated with a moderate certainty of evidence in terms of BoP% reduction (3 months) and HbA1c reduction (3 months), while the adjunctive use of DL was associated with a moderate certainty of evidence both for GI reduction (3 months) and for HbA1c reduction (3 months).

## Discussion

The results of the present systematic review and meta-analysis demonstrated a small but significant positive effect of the application of PDT as an adjunct to NSPT in type II diabetic patients regarding PD changes (6 months) and HbA1c changes (3 months) compared to control groups (NSPT only), the latter reporting more favorable CAL changes (6 months). Moreover, LT with DL as an adjunct to NSPT resulted in an enhanced effect for PD, CAL, GI, and HbA1c reductions at 3 months. However, these results need to be interpreted with caution due to the small effect sizes and the relatively high statistical heterogeneity.

Different from several other published systematic reviews that addressed mainly glycemic control [15, 16, 47, 48], the main focus of this systematic review was on post-treatment periodontal. As a matter of fact, our primary outcomes included the percentage of closed pockets, PD reduction, and CAL gain. Therefore, the results of the present research should be interpreted in the light of the existing literature that examined the same outcomes.

PDT was described as an effective antimicrobial strategy towards periodontal pathogens, and its activity depends on the creation of components that are noxious for the microorganisms (such as free radicals) following the activation, by the laser light, of the photosensitive component [49, 50]. Several laser types and applications were described as an adjunct for the treatment of periodontal diseases [51]. The rational of using laser for the treatment of periodontal pockets relates to the decontamination ability of the affected sites, particularly in situation of difficult access [52]. Moreover, laser application could result in accelerated healing and homeostasis, thus potentially improving the treatment outcomes [52].

Regarding the available evidence on the use of LT or PDT as an adjunct to NSPT, the systematic review published by Salvi and coworkers in 2020, using strict inclusion criteria, evaluated a total of 18 papers, of which only 2 could be included in the quantitative synthesis [20]. Their meta-analysis revealed a non-significant beneficial effect of PDT as an adjunct to NSPT in terms of PD changes [20]. Another systematic review about the application of LT for the management of untreated periodontitis and that performed meta-analysis on five papers did not suggest any significant effect on CAL or PD changes as well as PROMS over time [53]. Other recently published papers have provided further data on the topic without solving the controversy, as both favorable results [54, 55] and clinically insignificant benefits [56] were suggested. The results of our meta-analysis, although showing a significant effect in some comparisons of PDT/LT + NSPT, failed to clearly demonstrate a clinically relevant beneficial effect, being coherent with the previously cited studies.

While all the aforementioned systematic reviews focused on systemically healthy subjects, Abduljabbar and coworkers aimed at exploring the role of lasers as adjunct to NSPT in subjects with diabetes [25]. The authors adopted different inclusion criteria than those considered in the present study, and included six articles in the final qualitative synthesis, three about LT and three about PDT, without presenting conclusive results [25]. Another review of the same group on PDT included four RCTs and concluded that no difference between test and control group could be observed in terms of clinical parameters [26]. Compared to the works by Abduljabbar et al., our research included a higher number of recent papers by using different inclusion criteria, thus presenting updated data on the topic. Moreover, we performed a risk of bias evaluation with standardized methods, and we included in the meta-analysis more outcome variables. Additionally, the present research included the evaluation of the quality of evidence, which should be considered a crucial aspect for weighting the validity of the results.

Another important aspect to consider when dealing with diabetic patients is the effect that periodontal treatment might have on glycemic control. A recent Cochrane systematic review on the improvements in glycemic control (measured by the HbA1c changes) in subjects treated with NSPT compared to controls indicated a decrease of 0.43% (CI95%: 0.28–0.59) of HbA1c in test group at 3–4 months, with positive results also in longer follow-ups [15]. Although our main aim was not to assess changes in diabetes control, our meta-analysis suggested an adjunctive effect of PDT on HbA1c after 3 months of 0.24% (CI95%: 0.17–0.32), which was not confirmed after 6 months. Remarkably, studies on the efficacy of other adjunctive treatments to NSPT in subjects with diabetes, such as systemic antibiotics, found no significant additional effects in terms of glycaemic control [57, 58]. The regression meta-analysis performed in the present review failed to reveal a significant effect of baseline HbA1c% on PD changes and CAL changes. However, it should be noted that the relatively low number of papers available for each outcome and for each comparison may have limited the reliability of such analysis. Nevertheless, the risk of bias evaluation revealed a substantially moderate quality of the included studies, being six studies at low risk of bias. We can therefore reasonably assume that the results of the meta-analysis and the quality appraisal of the evidence were not affected by bias.

It is worth to acknowledge that the present systematic review had few shortcomings, as this might help to better consider the validity of the results and to interpret its findings. First, we should highlight that a substantial heterogeneity existed among the included study protocols regarding the characteristics of diabetes and the level of glycaemic control, the ethnicity of the population, the settings of the laser device, and the characteristic of periodontitis (namely severity), and this was probably the main cause of the statistical heterogeneity in the meta-analysis. Moreover, very limited data were available about the proportion of pocket closure, which is considered the most reliable outcome when evaluating the results of NSPT [59]. The lack of data about this outcome is a limiting factor, although PD and CAL changes are surrogate outcomes widely accepted and reported in the literature [60].

On the other hand, one strength of the present review is that to the best of our knowledge, this is the first systematic review on periodontitis and diabetes that also assessed the certainty of evidence for all the comparisons and outcomes included in the meta-analysis based on GRADE. The GRADE is a well-recognized tool for weighting the level of evidence of assumptions derived from a study, ideally a systematic review, in order to provide also clinical recommendations [32]. The GRADE is now fully integrated in Cochrane systematic reviews [27]; however, it is not frequently adopted in systematic reviews in the field of dentistry. In the authors’ opinion, considering the level of evidence and combining it with the statistical significance and the effect size can better inform on a clinically relevant topic such as the efficacy of PDT/LT. This comprehensive approach should be implemented whenever recommendations or clinically oriented guidelines are produced.

Finally, while it was not within the remit of this review to assess the cost-effectiveness of LT and PDT, the extra costs associated with the purchase and use of these physical therapies should be taken into account when considering whether or not to implement them in clinical practice and future studies are warranted to investigate the cost-effectiveness of these therapies.

In conclusion, taking all the aforementioned limitations into consideration, our review suggested that there is currently insufficient scientific evidence (and limited clinical relevance) to suggest the routine use of PDT or LT as an adjunct to NSPT in subjects with type II diabetes, although the promising results in terms of HbA1c decrease in the short term should be further explored in well-designed RCTs with > 6-month follow-up. It is recommended that future studies should consider the percentage of pocket closure as a primary outcome and explore the role of patient-reported outcome measures. It is also important that future studies will apply standard definitions of diabetes.

## Data Availability

The data supporting the findings of this study are available on request from the authors.

## Appendix 1. Search strategy

Tables 4, 5, and 6.

**Table 4.** MEDLINE via OVID

	MeSH term	Free-Text search
Population	Exp Periodontal Diseases OR alveolar bone loss/ AND Exp diabetes mellitus, Type 2 OR exp hyperglycemia	Periodontit* OR Parodontos$s OR periodontal disease OR pyorrhea OR Pericementit* OR gum disease OR (Periodont* ADJ2 pocket*) OR (Periodont* ADJ2 defect*) OR (Periodont* ADJ2 atroph*) OR (periodontal attachment ADJ2 loss) OR (periodontal bone ADJ2 loss*) OR (periodontal ADJ2 resorption*) OR furcation OR (alveolar bone ADJ2 loss) AND diabet* or DM2 OR NIDDM or hyperglyc*

The limitation to human studies was performed following the double negation strategy suggested by the Cochrane handbook, i.e. combining the results with NOT (exp animals/ not humans.sh.).

In addition, the following filters were applied:

• Filter to exclude systematic reviews:

NOT (((systematic OR state-of-the-art OR scoping OR literature OR umbrella) ADJ (review* OR overview* OR assessment*)) OR "review* of reviews" OR meta-analy* OR metaanaly* OR ((systematic OR evidence) ADJ1 assess*) OR "research evidence" OR metasynthe* OR meta-synthe*).tw. OR systematic review/ OR "systematic review (topic)"/ OR meta analysis/ OR "meta analysis (topic)"/

• Filter to exclude case reports and other non-relevant publications:

NOT (letter/ OR editorial/ OR news/ OR exp historical article/ OR anecdotes as topic/) OR (letter OR comment*.ti) ORcase reports/

• Filter to exclude guidelines:

(clinical adj3 pathway).ti,ab,kw. or (clinical adj3 pathways).ti,ab,kw. or (practice adj3 parameter).ti,ab,kw. or (practice adj3 parameters).ti,ab,kw. or algorithms/ or care pathway.ti,ab,kw. or care pathways.ti,ab,kw. or clinical protocols/ or Consensus/ or Consensus Development Conference.pt. or Consensus Development Conference, NIH.pt. or Consensus Development Conferences as Topic/ or Consensus Development Conferences, NIH as Topic/or critical pathway/ or guidance.ti,ab. or guideline*.ti. or guidelines as topic/ or practice guidelines as topic/ or Health Planning Guidelines/ or practice guideline/

**Table 5.** Cochrane library via CENTER

	**MeSH term**	**Free-Text search**
Population	Exp Periodontal Diseases OR exp alveolar bone loss AND Exp diabetes mellitus, Type 2 OR exp hyperglycemia	Periodontit* OR Parodontos$s OR periodontal disease OR pyorrhea OR Pericementit* OR gum disease OR (Periodont* NEAR/2 pocket*) OR (Periodont* NEAR/2 defect*) OR (Periodont* NEAR/2 atroph*) OR (periodontal attachment NEAR/2 loss) OR (periodontal bone NEAR/2 loss*) OR (periodontal NEAR/2 resorption*) OR furcation OR (alveolar bone NEAR/2 loss) AND diabet* or DM2 OR NIDDM or hyperglyc*

**Table 6.** EMBASE

	EMTREE terms	Free-Text search
**Population**	Exp Periodontal Disease OR alveolar bone loss AND Exp non insulin dependent diabetes mellitus OR exp hyperglycemia	Periodontit* OR Parodontos$s OR periodontal disease OR pyorrhea OR Pericementit* OR gum disease OR (Periodont* ADJ2 pocket*) OR (Periodont* NEAR/2 defect*) OR (Periodont* NEAR/2 atroph*) OR (periodontal attachment NEAR/2 loss) OR (periodontal bone NEAR/2 loss*) OR (periodontal NEAR/2 resorption*) OR furcation OR (alveolar bone NEAR/2 loss) AND diabet* or DM2 OR NIDDM or hyperglyc*

The results were limited to human studies, clinical studies, articles and articles in press with the dedicated EMBASE limits.

## Appendix 2. Procedure for calculating SD

For each presented outcome, the difference between baseline and follow-up values were extracted (with specific error measure such as standard deviation (SD) or standard error (SE) or variance). When such parameter was not presented, it was computed as the difference between baseline and follow-up values. In these cases, following the instructions of the Cochrane Handbook for Systematic Reviews when SDs of changes values were not presented and they were not provided by authors after contacting them by email, they were computed as follows: i) if similar studies were present (similar treatment, similar population, similar sample size), SD was imputed taking the value of the other study; ii) when *P* value is presented SD was computed by using T tables for retrieving SEs; iii) when *P* value is presented as a limit (e.g. < 0.05) a conservative value of *P* (e.g. 0.05 in case of < 0.05) was considered for computing SE as described before; iv) if *P* value was not present SDs of change values was imputed by using the following formula [27, 61, 62]:

$$SDcv=\sqrt{SDbaseline^2+{SDfinal}^2-\left(2\ast CORR\ast SDbaseline\ast SD\;final\right)}$$

being CORR the correlation coefficient, that could be imputed from similar studies if present, or it was assumed conservatively to be 0.2. For each measure, pooled estimate of 95% CI was calculated.
